# Adaptation and validation of the Brazilian version of the Measure of Moral Distress for Healthcare Professionals (MMD-HP BR) in the context of palliative care

**DOI:** 10.1186/s12904-023-01277-3

**Published:** 2023-10-11

**Authors:** Julianna Rodrigues Beltrão, Marianna Rodrigues Beltrão, Rafaella Stradiotto Bernardelli, Renato Soleiman Franco, Elizabeth G. Epstein, Carla Corradi-Perini

**Affiliations:** 1 Pontifícia Universidade Católica do Paraná, Rua Imaculada Conceição, Curitiba, 80215-901 PR Brazil; 2 University of Virginia School of Nursing, Charlottesville, VG USA

**Keywords:** Bioethics, Moral distress, Palliative care, Healthcare professionals, Validation studies

## Abstract

**Background:**

The Measure of Moral Distress for Health Care Professionals (MMD-HP) scale corresponds to the update of the globally recognized Moral Distress Scale–Revised (MDS-R). Its purpose is to measure moral distress, which is a type of suffering caused in a professional prevented from acting according to one’s moral convictions due to external or internal barriers. Thus, this study has the objective to translate, culturally adapt, and validate the Brazilian version of the MMD-HP BR in the context of Palliative Care (PC).

**Methods:**

The study had the following steps: translation, cross-cultural adaptation and validation. The MMD-HP BR is composed of 27 Likert-rated items for frequency and intensity of moral distress. In total, 332 health professionals who work in PC participated in the study, 10 in the pre-test stage, and 322 in the validation stage.

**Results:**

It was possible to identify six factors, which together explain 64.75% of the model variation. The reliability of Cronbach’s alpha was 0.942. In addition, the score was higher in those who are considering or have already left their positions due to moral distress, compared to those who do not or have never had such an intention.

**Conclusions:**

MMD-HP BR is a reliable and valid instrument to assess moral distress in the PC context. It is suggested that the scale be standardized in other healthcare contexts, such as clinical settings. In addition, further research on moral distress is encouraged to identify and reduce the phenomenon and its consequences.

**Supplementary Information:**

The online version contains supplementary material available at 10.1186/s12904-023-01277-3.

## Background

Moral distress is the term used to describe the experience of professionals who are prevented, by external and internal barriers, from acting based on their moral values or what they believe to be ethically correct in any context of health assistance [1]. Jameton, a philosopher, first described the phenomenon in 1984. The author characterized situations in which nurses performed actions judged to be morally wrong due to institutional pressures [2].

The effects of moral distress can vary, but, generally, they threaten the moral integrity of the subject who suffers. The manifestations may be directed at the professional, colleagues, or even the work institution, and include feelings of anger, frustration, self-depreciation, and sadness. In addition, they are related to burnout syndrome and the intention to leave the job [3, 4].

Palliative care [PC] consists of the assistance provided by a multidisciplinary team to patients and their families in the face of a life-threatening disease, to improve the quality of life [5]. Therefore, acting in this area involves daily ethical issues regarding the limits between life and death. As a consequence, health professionals are at greater risk of suffering from moral distress [6]. Therefore, this research focused on health professionals who work in palliative care.

In the context of PC, four categories of barriers causing moral distress were identified, namely: patient and family, team, organization, and personal factors. Among the main situations, the following stand out: communication failures; fear of peer judgment; lack of resources and professionals; dysthanasia, and other treatments considered “futile” and that cause suffering to the patient [7, 8].

Studies in the area have led to the development of tools to measure moral distress in various contexts [9]. The most used and validated in other countries is the “Moral Distress Scale-Revised” [MDS-R] scale, which is based on three categories of causes for the phenomenon: clinical situations; internal barriers; and external. In 2019, Epstein and colleagues adapted it to a new version, called “Measure of Moral Distress for Healthcare Professionals” [MMD-HP]. The new version adds excessive documentation factors and administrative directives to the impact on the quality of care provided and has already been adapted for use in Japan and Sweden [10–12].

In Brazil, the MDS-R was translated and validated for application in nurses by Ramos and colleagues [13]. However, publications on this topic in the country are still incipient and focused only on the nursing profession.

Given the relevance of the topic, it is essential to validate instruments that assess the occurrence and impact of moral distress, contributing to the development of intervention proposals to mitigate the phenomenon. That being the case, the present study aims to translate and adapt the MMD-HP scale to Portuguese-BR and validates the instrument among health professionals working in PC in the Brazilian scenario.

## Method

This is a cross-sectional, exploratory, analytical study with a quantitative approach. The research was performed in accordance with the Declaration of Helsinki and was approved by the Research Ethics Committee at Pontifícia Universidade Católica do Paraná (PUCPR), under CAAE nº 31397220.5.0000.0020. All participants agreed with the information in the informed consent form. In addition, the main author of the scale was contacted by e-mail and granted authorization to conduct the research.

### Instrument

The original version of the MMD-HP is composed of 27 situations of potential moral compromise and each item includes a five-point Likert score for frequency and level of distress. Responses range from 0 - never/null to 4 -high/very high. At the end of the items, there is an open question for the participant to list any other situation causing moral distress. In addition, two questions assess the intention to leave the job due to current or past moral distress. The scale does not have domains and, therefore, must be analyzed globally [10].

To score responses, the frequency score and distress level of each item must be multiplied, resulting in a value between 0 and 16 points. This scoring approach results in the elimination of items never experienced or not seen as distressing. Subsequently, the score for each item must be added, resulting in a total ranging from 0 to 432 points. According to the interpretation, higher the score, greater the moral distress [10].

The open-ended option to describe a situation causing moral distress, contained at the end of the questionnaire, is not included in the score and is mainly used to monitor new causes of moral distress not included in the instrument [10]. The instrument applied to the participants of this research also had socio-demographic questions and the characterization of the work in PC.

### Participants

Inclusion criteria: health professionals - with higher or technical education concluded -, of any age group and of both sexes, who work within the scope of PC in locations within the national territory. There are no exclusion criteria.

### Translation and adaptation

To translate and culturally adapt the MMD-HP, six steps were followed [14], aimed at semantic, idiomatic, experiential, and conceptual adaptation. The steps were: initial translation; synthesis of translations; retranslating; expert committee; pre-test; and review of the adaptation process by the researchers.

In the initial translation, the instrument was sent to two independent bilingual translators to translate from English to Portuguese. One of the translators was aware of the objectives and concepts used in the scale, while the other was unaware of any information regarding the subject of the study. Subsequently, a synthesis of the two translations was elaborated, containing the verified discrepancies and their solutions. Afterward, two other translators, who were not informed about the contents and objectives of the instrument, retranslated this version into English. At this stage, the authors verified possible discrepancies between the two English version and the original version of the scale and which words in Portuguese-BR that could have caused such differences. After modifications by the authors, the last version was sent to a committee of Bioethics and PC experts composed of four professionals for opinions, formulating the pre-final version.

A pre-test took place using the Portuguese-BR version, involving a sample comprising 10 healthcare professionals who work in PC, aiming to guarantee the validity of the instrument’s content. The pre-test population included professionals from the following areas: Medicine, Nursing, Nutrition, Psychology, and Physiotherapy who work in PC. The application took place individually so that each participant could report their difficulties and facilities while filling it out. The criterion established for reviewing and modifying the translation was the understanding of the items by less than 80% of the interviewees or the suggestion of modifications by more than 20% of the respondents. In the pre-test, the scale was well understood, the suggestions mentioned were: repetition of the word “experienced” in the instructions; specifying whether the answers should be about past or present performance; replacing the word “thank you”; among others.

Finally, the last stage was the review of the adaptation process by the researchers, who made the necessary modifications to facilitate the understanding of the instrument. The resultant scale was nominated “the Measure of Moral Distress for Healthcare Professionals - Brazilian version” [MMD-HP BR] [supplementary file].

### Validation

A free and informed consent form, the final version of the scale, and identification questions were self-administered, online, with the aid of the *Google Forms* platform. The convenience sample should have at least 270 health professionals working in PC in Brazil. It is noteworthy that there is no determination of the sample size related to the psychometric validation of instruments, but it is estimated that the sample is composed of the rule of 10 participants per question of the questionnaire [12]. Content validity was evaluated through the pre-test and adaptation in the translation stage.

### Statistical analysis

An exploratory factor analysis was performed to describe the underlying structure of the MMD-HP. First, the partial correlations of the 27 questions were tested using the Kaiser-Meyer-Olkin [KMO] statistics; as a criterion for adopting the factor analysis model, a KMO statistic value greater than 0.8 was considered an adequacy criterion for the use of factor extraction, using a principal component analysis [PCA] to assess their number. To interpret the factor loadings, there is a general rule that when the question has a factor loading greater than or equal to 0.7, it corresponds to about half of the variance in the indicator being explained by the component/factor. However, the 0.7 standard is high and real-life data may not meet this criterion. This is why some researchers, especially for exploratory purposes, will use a lower level such as 0.4 for the factor. Some authors call loads above 0.6 “high” and those below 0.4 “low”. In any case, factor loadings must be interpreted in the light of theory, not just arbitrary cut-off levels, i.e., it has to make clinical sense [15, 16]. The identified factors were named by the authors in consensus, considering the literature on the subject and similar previous studies.

Principal component analysis was performed using the scree plot through the Elbow rule and factor loadings greater than 0.4 [in modulus] were considered. The reliability of the score was evaluated by internal consistency using Cronbach’s alpha, with a minimum value of 0.70 as desirable [17].

## Results

**Fig. 1:**
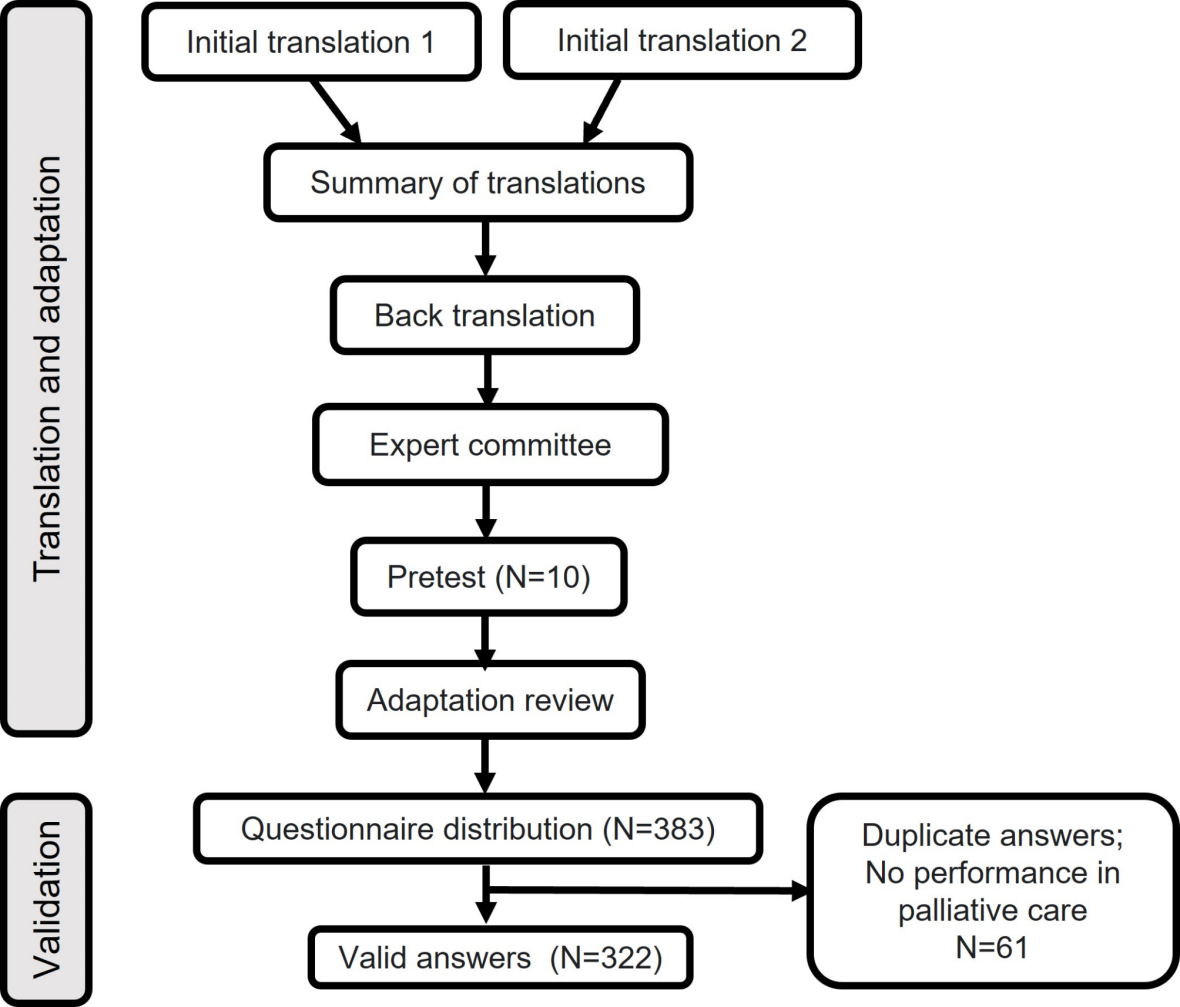
Stages of the study. Legend: After the distribution of the final questionnaire, 383 responses were received, of which 61 were excluded because they were from professionals who do not work in PC or due to repetition, resulting in 322 valid responses

The stages of the present study are represented in Fig. 1.

### Principal components and Associations Analysis

#### Characterization of participants

Personal, professional, and sociodemographic characteristics are shown in Table 1. The mean age of the participants was 38 years, and the majority of the sample was female [85.4%]. The professions with the highest participation were Medicine, Psychology, and Nursing.

**Table 1:** Description of sample (n = 322)

Variables	Results
*Age in years, average ± DP (min-max.)*	38 ± 9.4 (21–78)
*Sex*	N (%)
Female	275 (85.4)
Male	47 (14.6)
*Profession*	N (%)
Physician	102 (31.7)
Psychologist	70 (21.7)
Nurse	63 (19.6)
Physical Therapists	32 (9.9)
Nutritionist	15 (4.7)
Speech Therapist	11 (3.4)
Nursing Technician	10 (3.1)
Social Worker	8 (2.5)
Pharmacist	4 (1.2)
Occupational therapist	4 (1.2)
Chaplain	2 (0.6)
Environmental Therapist	1 (0.3)

### Construct validity

An exploratory factor analysis was performed to describe the underlying structure of the MMD-HP BR, in which all 322 responses were considered, as there was no missing data in any of the questions.

The result of the KMO showed excellent global adequacy of the 27 items of the MMD-HP BR with the magnitudes of the partial correlations of 0.938. Thus, PCA was adopted from the factor extraction.

How much each of the instrument’s questions is correlated with the others, that is, its commonality [h^2^] is expressed in Table 2, which is shown that the higher the commonality value, the greater the percentage of variance in a given variable explained by all factors together.

**Table 2:** Commonality of questions

Question	Commonality (h^2^)
1	0.538
2	0.635
3	0.642
4	0.496
5	0.699
6	0.664
7	0.645
8	0.622
9	0.606
10	0.582
11	0.646
12	0.659
13	0.565
14	0.705
15	0.584
16	0.721
17	0.779
18	0.698
19	0.699
20	0.674
21	0.637
22	0.535
23	0.754
24	0.693
25	0.686
26	0.721
27	0.598

**Fig. 2:**
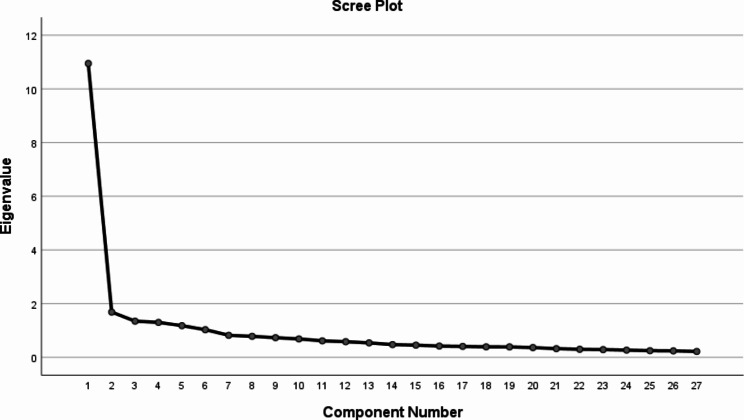
Scree Plot of factorial analysis of principal components. Legend: The most abrupt drop in the curve demonstrates the great weight of only one component, which even stands out for explaining 40.54% of the model alone

Figure 2 represents the Scree Plot of the factor analysis of the principal components. Based on the Kaiser criteria six factors were identified, which together explain 64.75% of the model variation. Among the factors, one stands out for explaining 40.54% of the model alone.

The questions for each factor and the respective factor structure when rotated using the Varimax Orthogonal Rotation are shown in Table 3. The highlighted values represent a substantial influence of the question on the component composition.

**Table 3:** Varimax rotation

Question	Components					
	1	2	3	4	5	6
17	**0.828**	0.081	0.044	0.045	0.156	0.243
18	**0.739**	0.293	0.174	0.109	0.140	0.066
14	**0.667**	0.263	0.326	0.195	0.212	0.046
9	**0.568**	0.122	0.280	0.326	0.287	0.023
24	**0.495**	0.443	0.270	0.323	0.239	0.135
15	**0.457**	0.333	**0.438**	0.024	0.126	0.236
1	**0.415**	0.069	**0.412**	0.379	0.205	0.071
26	0.256	**0.677**	0.179	0.314	0.233	0.111
27	0.330	**0.666**	0.082	0.109	0.010	0.164
21	0.031	**0.661**	0.269	0.142	0.307	0.112
25	0.325	**0.596**	0.112	0.237	0.383	0.098
20	0.035	**0.557**	0.378	0.192	0.380	0.196
6	0.138	0.233	**0.734**	0.105	0.009	0.202
11	0.296	0.356	**0.645**	0.109	0.053	0.016
4	0.186	0.057	**0.589**	0.169	0.285	0.036
13	0.376	0.388	**0.452**	0.126	0.220	0.073
5	0.046	0.222	0.173	**0.760**	0.094	0.176
8	0.062	0.217	0.003	**0.715**	0.015	0.246
2	0.204	0.030	0.142	**0.701**	0.219	0.182
3	0.293	0.315	0.224	**0.601**	0.126	0.171
12	0.048	0.294	**0.420**	**0.471**	0.098	**0.402**
19	0.249	0.228	0.030	0.111	**0.743**	0.140
23	0.174	0.366	0.077	0.104	**0.742**	0.149
16	**0.436**	0.016	0.175	0.075	**0.563**	**0.421**
22	0.180	0.267	0.311	0.117	**0.562**	0.063
10	0.066	0.065	**0.497**	0.262	**0.498**	0.100
7	0.160	0.049	0.124	0.226	0.232	**0.704**

The result of the KMO showed excellent global adequacy of the 27 items of the instrument, with magnitudes of partial correlations of 0.938.

Regarding the factors and their significations, it can be concluded that: factor 1 indicates the interaction between professionals and patients or family members, in which are noted lack of communication; factor 2 is the relationship between the professional and their co-workers; factor 3 personal-pressures; factor 4 therapeutic obstinacy; and factor 5 system/hospital.

The reliability of the instrument and six components was evaluated by measuring the internal consistency using Cronbach’s alpha. The results are shown in Table 4 and demonstrate high internal consistency. Component 6 is composed of only one question, so it was not tested.

**Table 4:** Internal Consistency

Component	Questions tested	Cronbach’s Alpha
MMD-HP BR	All (1–27)	0.942
1	1, 9, 14, 15, 17, 18 e 24	0.879
2	20, 21, 25, 26 e 27	0.842
3	4, 6, 11 e 13	0.761
4	2, 3, 5, 8 e 12	0.809
5	10, 16, 19, 22 e 23	0.811
6	7	

### Correlation and association measures – convergent validity

Based on the results presented in Table 5, it appears that there is a significant difference in the value of the score between the groups to questions of abandonment of the previous work position [p = 0.001] and currently [p = 0.001]. Professionals who have already considered leaving, have already left, or are currently considering leaving, have higher moral distress scores compared to those who have never left and are not considering leaving their job.

**Table 5:** Comparison of the score between the groups concerning leaving the job

Question	Group answer	n	Score (average; med. (min-max.)	The p-value of the global comparison*	Adjusted p-value from the comparison between groups 2 to 2**
Have you ever left or considered leaving a clinical position due to moral distress?	No, I have never considered leaving or left a position	106	66.1; 53 (0–296)	< 0,001	No vs. Yes, but did not leave p < 0.001
	Yes, I considered leaving but did not leave	136	119.5; 97.5 (12–376)		No vs. Yes, left a position: p < 0.001
	Yes, I left a position	80	126.8; 114.5 (0–387)		Yes, but did not leave vs. Yes, left a position: p < 0.001
Are you considering leaving your position now due to moral distress?	No	253	91.6; 73 (0–314)	< 0.001	
	Yes	69	148.3; 136 (7–387)		

## Discussion

Health professionals who work in PC are in daily contact with patients with limited life expectancy and cases characterized by ethical and moral conflicts. This exposure makes them more vulnerable to suffering from moral distress [6].

In the context of pediatric and neonatal PC, a systematic review showed that the moral distress of health professionals was related to communication problems, family denial of the disease, futile or disproportionate treatments, false hopes, ineffective management of symptoms, and the disagreement between the team about the best course of action [18]. These situations are present in the scale applied in the present study.

In the present study, six factors were identified. This is a different finding from several previous studies. The authors hypothesize that this difference may be due to the specific context of PC, which commonly involves uncertainties in decision-making about end-of-life care. These decisions draw fine lines between therapeutic obstinacy and the adequacy of care, less observed in other health contexts [7].

In the development of the original MMD-HP, the situations causing distress were analyzed in the questionnaire in four main factors, namely: system, clinician/patient, team/staff, and team/patient. The team factor refers to two situations: causes related to commitments to individual integrity; and those linked to interactions between professionals and patients or family members [10].

A four-factor structure was also found in the adaptation of the instrument in the Japanese cohort. The study also showed that the adjustment in the model for three factors was similar. However, the three-factor structure with 22 items should not replace the original model [12]. Another division of the causes of moral distress was proposed by Maffoni and colleagues [8], in which four categories are identified: personal factors; patients and family members; colleagues and superiors; environment, and organization.

Professionals who have already considered, left, or are currently considering leaving their jobs had significantly higher levels of moral distress. This correlation is well established in the literature [19, 20] and may be related to the increasing effect described by Epstein and Hamric [21], according to which repeated experiences with moral distress lead to a progressive increase in suffering.

In addition to the intention to leave the job and emotional withdrawal, moral distress is also related to the occurrence of burnout [20, 22–24], which can compromise the quality of care offered to the patient and his family and the moral integrity of the subject who suffers. Thus, although interventions to prevent or reduce moral distress are still being developed, it is believed that they could bring tangible benefits, such as job retention and better teamwork [25].

### Limitations

This study has several limitations, even with the necessary adaptations made. Due to the origin of the questionnaire, developed in the United States, where the culture is different, some aspects may not represent the Brazilian reality. The choice of participants in the palliative care context itself is a limitation. Therefore, regarding the sample size, it was noted that some professions had low representation, which may have impacted the analysis of certain indicators. Although the results presented are consistent with the literature on the subject and studies carried out, the data may be biased and difficult to generalize. Finally, there is also the inconsistency of research on moral distress, especially in Brazil, which may have hampered some analyses.

## Conclusions

From the process of translation, adaptation, and validation of the MMD-HP BR, it can be concluded that the instrument showed good results of reliability and construct validity in the assessment of moral distress of health professionals who work in PC.

It is suggested that a broader validation should be carried out in different environments and professional contexts, to confirm the psychometric properties of the Brazilian version of the MMD-HP. Other possible causes of moral distress, specific to the Brazilian population, should also be investigated. In addition, the consolidation of the term in the Brazilian context is indicated, allowing new studies to contribute to the investigation and reduction of the phenomenon and its consequences.

### Electronic supplementary material

Below is the link to the electronic supplementary material.

Supplementary Material 1

## Data Availability

The datasets used and/or analysed during the current study are available from the corresponding author on reasonable request.

## List of abbreviations

PC: Palliative care
MDS-R: Moral Distress Scale-Revised
MMD-HP: Measure of Moral Distress for Healthcare Professionals
MMD-HP BR: Measure of Moral Distress for Healthcare Professionals – Brazilian version
KMO: Kaiser-Meyer-Olkin
PCA: Principal component analysis
