# Supplementary material for: Adaptation and validation of the Brazilian version of the Measure of Moral Distress for Healthcare Professionals (MMD-HP BR) in the context of palliative care
Source: BMC Palliat Care. 2023 Oct 11;22:154. doi: 10.1186/s12904-023-01277-3 (PMC10566136; doi:10.1186/s12904-023-01277-3)
Supplement: Supplementary file 1 — Supplementary Material 1 [file 12904_2023_1277_MOESM1_ESM.pdf]

MEASURE OF MORAL DISTRESS FOR HEALTHCARE PROFESSIONALS – VERSÃO BRASILEIRA (MMD-HP BR)

O distresse moral ocorre quando os profissionais não conseguem atuar de maneira que consideram apropriada e ética devido barreiras ou restrições externas ou internas. Esta pesquisa lista situações que podem ocorrer na prática clínica.

Se você vivenciou alguma destas situações, ela pode ou não ter sido moralmente angustiante a você. Por favor, indique a frequência com que experienciou cada item, em uma escala de 0 (Nunca) a 4 (Muito Frequente). Além disso, assinale o quão angustiante essas situações foram para você (Nível de Distresse Moral), em uma escala de 0 (Nenhum) a 4 (Muito angustiante). Caso você nunca tenha experienciado a situação em particular, assinale "0" (nunca) para frequência. Mesmo se não tiver passado pela situação, por favor indique o quão angustiante seria caso viesse a ocorrer em sua prática profissional.

Observe que você deverá responder a cada um dos itens para as duas dimensões: *Frequência da ocorrência e Nível de Sofrimento.*

	**Frequência**					**Nível de Distresse**				
	**Nunca**				**Muito**	**Nulo**		**Muito Elevado**		
	0	1	2	3	4	0	1	2	3	4
1. Observo profissionais de saúde comunicando "falsas esperanças" ao paciente ou família.										
2. Aceito a insistência da família para continuar com tratamentos / intervenções invasivos mesmo que eu acredite que não é o melhor para o paciente.										
3. Me sinto pressionado para solicitar ou executar exames ou tratamentos que eu considero desnecessários ou inapropriados.										
4. Sou incapaz de ofertar o cuidado que julgo ideal devido às pressões de administradores ou dos planos de saúde para reduzir custos.										
5. Continuo a fornecer tratamento invasivo para um paciente em fim de vida e que provavelmente irá falecer independente deste tratamento, enquanto ninguém tome a decisão para suspendê-lo.										
6. Sou pressionado a me calar quando algum colega / membro da equipe cometeu um erro e não o reportou.										
7. Devo cuidar de pacientes, os quais eu não me sinto qualificado para atender.										
8. Realizo procedimentos que causam sofrimento desnecessário ou que não são adequados para aliviar dor ou outros sintomas desconfortáveis do paciente.										
9. Presencio um paciente sofrer por conta da falta de continuidade do tratamento.										
10. Atendo as orientações de um outro profissional da equipe ou de um membro da família de não informar o prognóstico ao paciente/família do paciente.										
11. Testemunho a violação dos protocolos de atuação dos profissionais da saúde ou código de ética e não me sinto apoiado o suficiente para reportar a violação.										
12. Participo de um cuidado ou procedimento que não concordo, mas o faço por receio de litígio (ser processado).										
13. Sou solicitado a trabalhar com outro membro da equipe que não é competente o suficiente para aquilo que o paciente demanda.										

14. Observo atendimentos de baixa qualidade devido à falta de comunicação entre os membros da equipe.										
15. Sinto-me pressionado a ignorar situações nas quais os pacientes não receberam informação adequada para garantir o consentimento informado.										
16. Sou solicitado a cuidar de mais pacientes do que tenho condições de atender de forma segura.										
17. Observo que o cuidado do paciente tem prejuízos devido à falta de recursos / equipamentos / leitos disponíveis.										
18. Presencio a falta de ações ou suporte administrativo para problemas que comprometem o cuidado do paciente.										
19. Tenho uma quantidade excessiva de tarefas burocráticas e documentação a ser preenchida que comprometem o cuidado do paciente.										
20. Tenho medo de retaliação caso eu me posicione.										
21. Sinto-me inseguro ou sofro "*bullying*" / julgamentos por parte de meus próprios colegas.										
22. Sou submetido a atender pacientes / familiares autoritários / inconvenientes que comprometem a qualidade do atendimento.										
23. Sinto-me exigido a dar prioridade a outras tarefas, medidas de produtividade ou de qualidade em detrimento do atendimento ao paciente.										
24. Sou solicitado a cuidar de pacientes com plano de tratamento inconsistente ou sem clareza nos objetivos do cuidado.										
25. Trabalho dentro de hierarquias em equipes, unidades e instituições que comprometem o atendimento ao paciente.										
26. Participo de uma equipe que fornece informações confusas ou contradizentes para o paciente/família.										
27. Trabalho com membros da equipe que tratam pacientes vulneráveis ou estigmatizados sem devido respeito e dignidade.										
28. Se há alguma outra situação na qual você sentiu distresse moral, por favor escreva e categorize abaixo:										

Você já abandonou ou considerou deixar uma posição de trabalho devido ao distresse moral?

- Não, nunca considerei sair ou deixar uma posição.
- Sim, já considerei sair, mas nunca saí.
- Sim, já deixei uma posição.

Você está considerando atualmente deixar sua posição de trabalho devido ao distresse moral?

- Sim.
- Não.
